# Evaluation of the Anticorrosion Performance of CeO_2_-Modified Graphene Oxide Nanocomposite Epoxy Coating Subjected to Simulated Saline-Alkali Solution

**DOI:** 10.3390/polym14071412

**Published:** 2022-03-30

**Authors:** Kai Lyu, Xiaoyan Liu, Ruidan Liu, Heng Yang, Yang Qiao, Surendra P. Shah

**Affiliations:** 1 College of Civil and Transportation Engineering, Hohai University, Nanjing 210024, China; 20200903@hhu.edu.cn; 2 Key Laboratory of Ministry of Education for Geomechanics and Embankment Engineering, Hohai University, Nanjing 210098, China; 3 College of Mechanics and Materials, Hohai University, Nanjing 211100, China; 201308030004@hhu.edu.cn; 4 Materials & Structural Engineering Department, Nanjing Hydraulic Research Institute, Nanjing 210024, China; hengyang@nhri.cn; 5 China Railway Shanghai Design Institute Group Co., Ltd., Shanghai 200070, China; qiaoyang@sty.sh.cn; 6 College of Engineering, University of Texas at Arlington, 701 S. Nedderman Drive, Arlington, TX 76019, USA; s-shah@northwestern.edu

**Keywords:** CeO_2_–GO, corrosion depth, saline-alkali, anticorrosion

## Abstract

In the marine service environment, metal materials have a serious risk of corrosion. The corrosion rate of metal materials will be accelerated by the dual action of temperature change and alkali salt in saline-alkali environment. In order to delay the metal materials’ corrosion rate and prolong their service life, this paper used a CeO_2_–GO (4:1) nanocomposite prepared by the hydrothermal synthesis method to make an anticorrosion coating. The anticorrosion performance was evaluated by stereo microscope and 3D images of the corrosion site were fitted for calculation. The state evolution of the CeO_2_–GO (4:1)/EP coating immerged in a simulated saline-alkali solution was studied by open circuit potential (OCP), electrochemical alternating current impedance spectroscopy (EIS), Mott–Schottky curve and Tafel curve. The results indicated that CeO_2_–GO (4:1) nanocomposites exhibited good resistance compared with graphene oxide and nano cerium oxide in a simulated saline-alkali environment. The research in this paper lays a firm theoretical foundation for the application of nano cerium-oxide-modified graphene oxide anticorrosive coating in saline-alkali environment engineering.

## 1. Introduction

Due to its superior physical properties, metallic materials are widely used in various infrastructure constructions. However, the main shortcoming of metallic materials is their corrosion resistance to aggressive species, and the corrosion can be seen on ships, bridges, base stations and even indoor metal equipment. Corrosion has brought hundreds of millions of economic losses to human society, and severely reduced the service life of components and equipment [1,2]. The corrosion of metal materials not only greatly shortens the life of components, but also causes a series of serious disasters. There are a lot of saline-alkali soil beside China’s long coastline and the vast northwest inland [3,4]. Saline-alkali soil is formed under certain natural conditions, and the essence of its formation is mainly the horizontal and vertical redistribution of various soluble salts on the ground, so that the salt accumulates gradually on the surface of the soil in the salt-collecting area [5,6]. Saline-alkali soil usually contains Cl^−^, Mg^2+^, SO_4_^2−^, Ca^2+^ and CO_3_^2−^. Among them, SO_4_^2−^ will corrode concrete and cause concrete expansion and cracking [7], and Cl^−^ will cause the corrosion of steel bars and various metal components in reinforced concrete [8]. Therefore, the development and preparation of anticorrosion materials is of particular importance.

In metal corrosion protection, the most commonly used method is surface coating. In coating anticorrosion, the most widely used and comprehensive effect is polymer coating. Polymer coatings generally possess superior chemical resistance, water resistance, strong adhesion to the substrate and easy curing properties. Therefore, polymer coatings are an appropriate choice for metal corrosion protection [9,10,11]. Epoxy resin (EP) is a kind of polymer material, which is produced by the polycondensation reaction of bisphenol A or polyol and epichlorohydrin. Epoxy resin is a kind of thermosetting resin with good mechanical strength and adhesion. However, during the curing process of epoxy resin, as the solvent continues to evaporate, micropores will be formed in the epoxy resin, and even some of the micropores will be transformed into interconnected holes [12,13]. Aziz et al. [14] studied the superhydrophobic modification of epoxy coating and provided a strategy to protect metals via simultaneously combining the protective functions of both superhydrophobic surface and organic protective coating. Chen et al. [15] prepared ZrO_2_-CNF/EP composite coating and showed that the corrosion current density decreased significantly with the addition of nanomaterials. Yang et al. [16] produced a sort of solvent-free transparent epoxy-modified silicone resin coating with high transparency and low volume contraction. It was also found that the obtained silicone materials had good thermal stability, good adhesive capacity and relatively low volume contraction. When epoxy resin is applied to the coating for anticorrosion, the corrosive medium continuously penetrates the substrate through the micropores, causing the substrate to corrode. Nanomaterials have a greatly specific outside area, which leads to a size effect at the same time. By incorporating nanocomposite materials to modify the anticorrosion coating, the above-mentioned problems can be better solved.

Graphene is a two-dimensional nanomaterial. Due to its two-dimensional-layered structure, compared to a rod and block structure, the layered structure has better barrier properties. Graphene oxide (GO) retains the layered structure of graphene, which provided the space for the graft of multiple functional groups to improve its chemical and physical properties. However, due to the inherent van der Waals force of graphene oxide, it is still difficult to disperse and stack between layers when it is incorporated into an epoxy resin. Therefore, it is possible to modify the nanoparticles between the layers of graphene oxide through chemical and physical reactions to improve its stacking characteristics. Yu et al. [17] prepared TiO_2_–GO/EP composite with epoxy resin and found that the addition of a TiO_2_–GO composite significantly improved the corrosion resistance of the coating when the content of composite is only 2 wt %. Cui et al. [18] modified GO nanosheets with hydrophilic dopamine (DA) to improve the easy agglomeration of GO by utilizing the π–π interaction between GO and self-polymerized polydopamine (PDA), as well as the covalent bond between DA and GO. Zhong et al. [19] prepared GO–Gr nanocomposite that increased the impedance modulus of the coating and slowed down the failure rate of the coating. Compared with the GO–EP coating, the GO–Gr/EP composite coating had better corrosion resistance. Jiang et al. [20] prepared GO slices with different length-width ratios and GO/EP coatings by controlling the oxidation degree of GO through a chemical stripping process and found that the anticorrosion performance of the corresponding coating would be better with the length-width ratio of GO.

Nano cerium oxide (CeO_2_) has a lamellar structure and can also enhance the anticorrosion ability of the coating [21,22]. In previous research [23], it was found that CeO_2_ was evenly distributed on the GO surface, which effectively reduced the agglomeration of GO. Moreover, when the mass ratio of CeO_2_ to GO was 4:1, the CeO_2_–GO (4:1)/EP nanocomposite coating had excellent anticorrosive performance. The study investigated the state evolutions of CeO_2_–GO (4:1)/EP coating during immersion in a simulated seawater solution with different concentrations and drew the conclusion that CeO_2_–GO (4:1)/EP coating also showed good anticorrosion performance [24].

In this paper, a CeO_2_–GO (4:1) nanocomposite was selected for preparing a CeO_2_–GO (4:1)/EP coating to compare with a pure EP coating, a GO coating and a CeO_2_ coating. The CeO_2_–GO (4:1) nanocomposite was prepared by a hydrothermal synthesis method [23]. The corrosion resistance of coated carbon steel in a simulated saline-alkali environment and temperature gradient cycle was investigated by electrochemical testing and microscopic characterization. The corrosion performance was observed by a stereo microscope and 3D images of the corrosion site were fitted. The state evolution of the CeO_2_–GO (4:1)/EP coating immerged in a simulated saline-alkali solution was studied by electrochemistry testing. The coating’s corrosion resistance and corrosion tendency were studied with the prolongation of immersion time in a simulated saline-alkali solution. The results are expected to further the application of cerium-oxide—graphene-oxide-modified anticorrosive coating in a saline-alkali environment.

## 2. Materials and Methods

### 2.1. Raw Materials

The graphene oxide (SE2430W) used in this research was a commercial product purchased from Changzhou Sixth Element Materials Technology Co., Ltd., Changzhou, China. The epoxy (WSR6101 E-44) and epoxy AB glue were supplied by Nantong Xingchen Synthetic Material Co., Ltd., Nantong, China. The cerium hexahydrate nitrate ((CeNO_3_)_3_·6H_2_O) was an analytical reagent obtained from Shanghai Aladdin Bio-Chem Technology Co., Ltd., Shanghai, China. The acetone (CH_3_COCH_3_) and ammonium hydroxide were supplied by Chengdu Colon Chemicals Co., Ltd., Chengdu, China. In the experiment, Q235 carbon steel with a size of 5 mm in height and 10 mm in diameter was selected to evaluate the anticorrosion performance of the developed coatings.

### 2.2. Preparation of Simulated Saline Solution and Temperature Gradient Cycle

The saline-alkali environment was complex. The carbon steel coated with the coating was placed in a simulated saline-alkali solution with a pH of 9 adjusted by NaOH. Table 1 shows the solute concentration in the simulated saline-alkali solution. The degree gradients were set at 5 °C, 25 °C and 45 °C, with a cycle every 36 h to simulate temperature fatigue of the coated carbon steel.

### 2.3. Preparation of CeO_2_–GO Epoxy Coating

**Figure 1:**
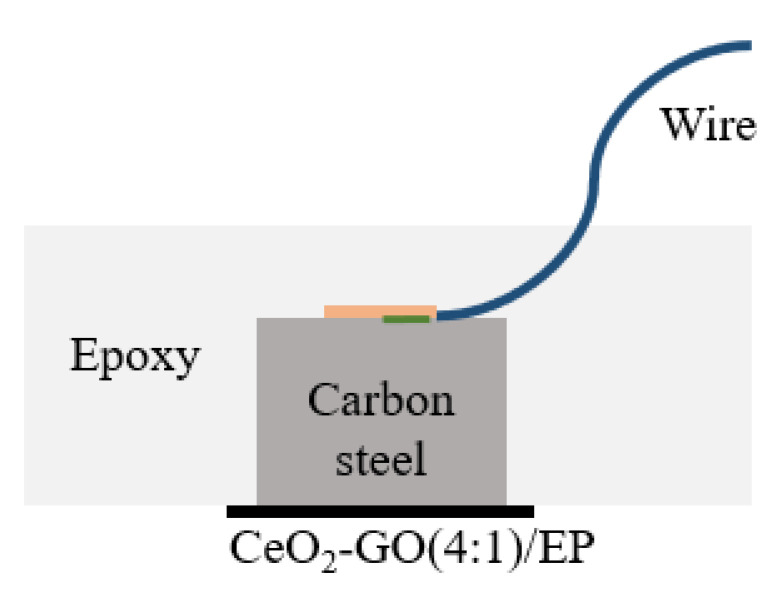
The coating sample schematic diagram.

The CeO_2_–GO (4:1) nanocomposite was prepared by hydrothermal synthesis method [23,24]. Figure 1 shows the schematic diagram of the preparation of the coating sample. The samples were placed in a simulated saline-alkali solution to investigate the effect of the coating. For comparison, four samples denoted as G1, G2, G3 and G4 were prepared, with G1 being the pure epoxy coating and G2, G3 and G4 being the epoxy coating with GO, CeO_2_, and CeO_2_–GO (4:1), respectively. The performance of G1, G2, G3 and G4 was compared to illustrate the anticorrosion resistance of the CeO_2_–GO (4:1)/EP coating in the simulated saline-alkali solution.

### 2.4. Testing Procedures

#### 2.4.1. Micromorphological Characterization

A HIROX-KH7700 stereomicroscope (Shanghai HIROX Instrument Technology Co., Ltd., Shanghai, China) was used to observe the corrosion of the substrate coated with each coating. The 3D morphologies of corroded parts on metal substrates were fitted by built-in software. The corroded areas of metal substrates were analyzed and calculated by contour maps of different depths, and the corrosion depth was calculated.

#### 2.4.2. Electrochemical Testing

An electrochemical workstation of PARSTAT 2273 type (AMETEK Group of Companies, Wilmington, MA, USA) was used for electrochemical testing, with a saturated calomel electrode as the reference electrode and a platinum electrode as the auxiliary electrode. The scanning range of the open circuit potential (OCP) was −200 Mv–1200 mV. The frequency range of EIS was 10^−2^–10^5^ Hz, and the amplitude was 10 mV. The scanning range of the potentiometric polarization curve was −300–300 mV, and the scanning rate was 1 mV/s. The adopted Mott–Schottky frequency was 1000 Hz, and the scanning interval was −1–0.5 V. The samples of G1, G2, G3 and G4 were tested.

## 3. Anticorrosion Performance

### 3.1. Open Circuit Potential (OCP) Analysis

**Figure 2:**
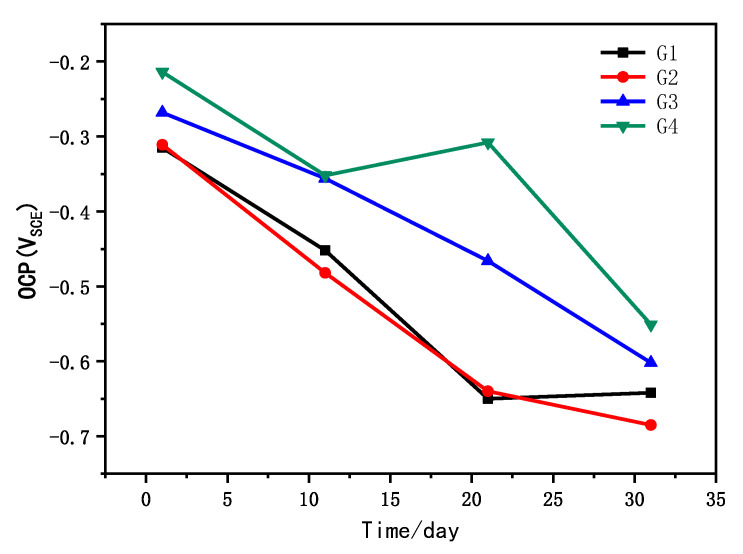
Each coating’s OCP after immersion for 31 d.

The open circuit potential is the potential difference of the working electrode and the reference electrode when there is no current flowing in the system, and the corrosion probability of the metal substrate can be judged by the open circuit potential. The four coatings’ OCP results corroded in the simulated saline-alkali solution for 31 d are shown in Figure 2. As we can see from the figure, G4 had the highest OCP value. A higher OCP value indicated that the coating had a lower corrosion sensitivity, that is, the higher the potential, the lower the corrosion trend. The OCP values of G4 at 1 d, 11 d, 21 d and 31 d were higher than those of other groups. The OCP value of the CeO_2_–GO (4:1)/EP coating was higher than that of the pure EP coating and the GO/EP coating, indicating that the nanocomposite had a better barrier effect and provided better protection for the metal matrix. Ce^4+^ itself has certain anticorrosion properties [21]. The incorporation of nanoparticles increases the compactness of the coating, and CeO_2_ has great compatibility with epoxy. It can be dispersed in epoxy and combined with epoxy. Meanwhile, it can be uniformly cemented with oxygen resin into a whole. However, the barrier properties of granular nanomaterials are not as good as layered nanomaterials, so their open circuit potential value was second only to CeO_2_–GO (4:1)/EP coatings. The two were relatively close and had similar corrosion tendency, indicating that if GO cannot be well dispersed in the epoxy, the weak points corrode, so that the corrosion occurs closer to the EP coating. When both of them were immersed for 11 d, the open circuit potential values were lower than −400 MV. Corrosion had already occurred on the substrate, and the protection performance was poor.

### 3.2. Electrochemical Alternating Current Impedance Spectroscopy (EIS) Analysis

**Figure 3:**
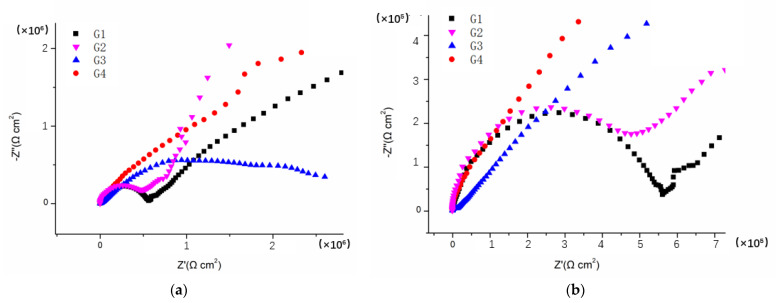
Nyquist patterns of G1, G2, G3 and G4 after immersion for 1 d: (**a**) Nyquist patterns at 1 d; (**b**) amplification of Nyquist patterns at 1 d.

**Figure 4:**
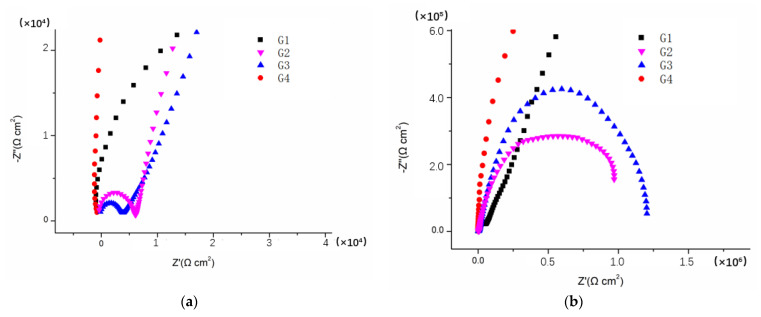
Nyquist patterns of G1, G2, G3 and G4 after immersion for 11 d: (**a**) Nyquist patterns at 11 d; (**b**) amplification of Nyquist patterns at 11 d.

**Figure 5:**
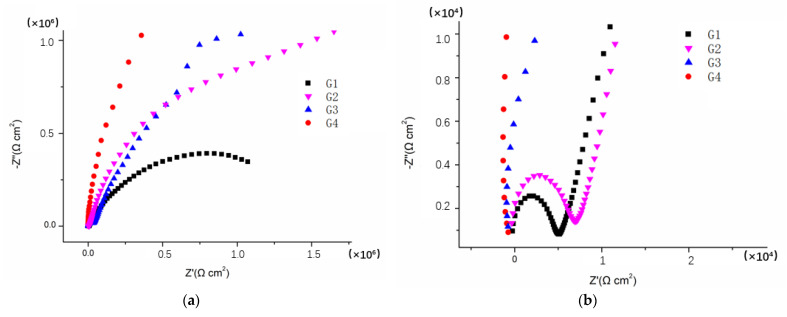
Nyquist patterns of G1, G2, G3 and G4 after immersion for 21 d: (**a**) Nyquist patterns at 21 d; (**b**) amplification of Nyquist patterns at 21 d.

**Figure 6:**
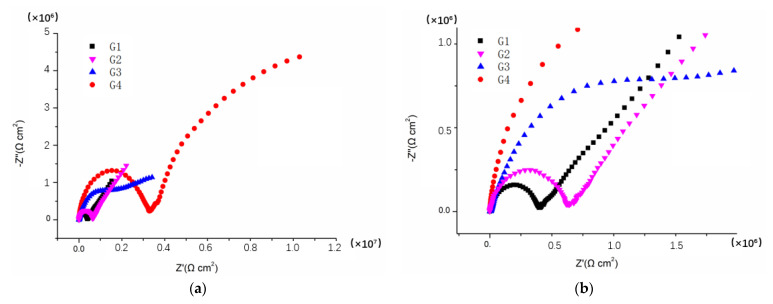
Nyquist patterns of G1, G2, G3 and G4 after immersion for 31 d: (**a**) Nyquist patterns at 31 d; (**b**) amplification of Nyquist patterns at 31 d.

Figure 3, Figure 4, Figure 5 and Figure 6 show Nyquist patterns of carbon steel coated with different coatings immersed in the saline-alkali solution for 1 d, 11 d, 21 d and 31 d. It can be seen from Figure 3 that after soaking for 1 d, the capacitive arc radius was large, and there was only one capacitive arc, which indicated that the coating was at the initial immersing stage and the barrier performance of each coating was intact, and no corrosive medium penetrated the coating to reach the metal matrix [25]. A complete capacitive arc appeared in G1 and G2, and the capacitive arc radius was smaller than G3 and G4, indicating that the CeO_2_–GO (4:1)/EP coating and CeO_2_/EP coating had better anticorrosion effects after immersion for 1 d. With the extension of the immersion time, the capacitive arc radius of each coating was continuously reduced. The capacitive arc radius of G1 and G2 was significantly reduced after immersion for 11 d, indicating that the corrosive medium continued to invade the coating. Although the capacitive reactance arcs of G3 and G4 decreased continuously, it still maintained a relatively large radius. With the continuous serious erosion, the capacitive resistance arc radius of each coating increased. This was because corrosion products’ accumulation on the surface of the substrate hindered and slowed down the further development of corrosion. Therefore, there would be an abnormal phenomenon of capacitive resistance arc. By combining with the Bode patterns, the anticorrosion performance of each coating was further analyzed [26].

**Figure 7:**
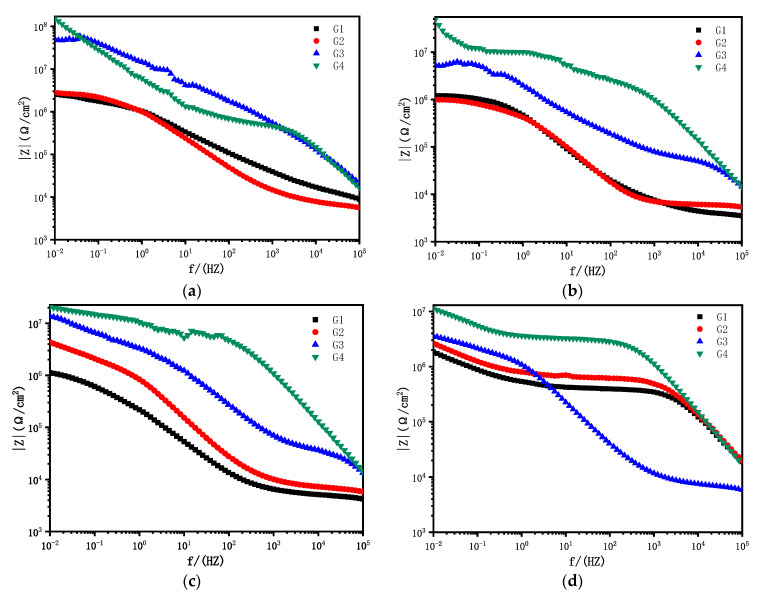
Each coating’s Bode pattern after immersion for 1 d, 11 d, 21 d and 31 d, respectively: (**a**) Bode pattern at 1 d; (**b**) Bode pattern at 11 d; (**c**) Bode pattern at 21 d; (**d**) Bode pattern at 31 d.

Figure 7 showed the Bode patterns of carbon steel coated with G1, G2, G3 and G4 immersed in the simulated saline-alkali solutions for 1 d, 11 d, 21 d, 31 d. The modulus value in the low-frequency region of the Bode pattern can reflect the protective performance of the coating. It can be seen from Figure 7 that the low-frequency impedance modulus of G4 reached 10^8^ Ω cm^2^ after immersion for 1 d. At this time, the coating had a good barrier effect, followed by G3, for which the low-frequency impedance modulus value reached 10^7^ Ω cm^2^, while the low-frequency impedance modulus value of G1 and G2 was 10^6^ Ω cm^2^. The low-frequency impedance modulus values of the four coatings were divided into three different orders of magnitude. With the prolongation of immersion time, the corrosive medium penetrated into the coating and reached the metal matrix to cause metal corrosion. The low-frequency impedance modulus of G1 decreased from 2.62 × 10^6^ Ω cm^2^ to 1.12 × 10^6^ Ω cm^2^, and then increased slightly in the later period. Although the decrease was not obvious, the low-frequency impedance modulus of the coating was always at a low level, which showed poor anticorrosion performance combined with the Nyquist pattern. G2 was similar to G1, and its low-frequency impedance modulus decreased from 2.81 × 10^6^ Ω cm^2^ to 9.82 × 10^5^ Ω cm^2^, with a slight increase in the later period, holding at the magnitude of 10^6^. The low frequency impedance modulus of G3 decreased from 5.02 × 10^7^ Ω cm^2^ to 4.39 × 10^6^ Ω cm^2^, indicating that its anticorrosion ability decreased obviously. The low-frequency impedance modulus of G4 decreased from 1.51 × 10^8^ Ω cm^2^ to 1.12 × 10^7^ Ω cm^2^, and the low-frequency impedance modulus at 31 d decreased by one order of magnitude compared with 1 d. However, it still remained at a high value, indicating that the coating’s anticorrosion performance was still optimal despite the erosion media invasion.

### 3.3. Tafel Curve Analysis

**Figure 8:**
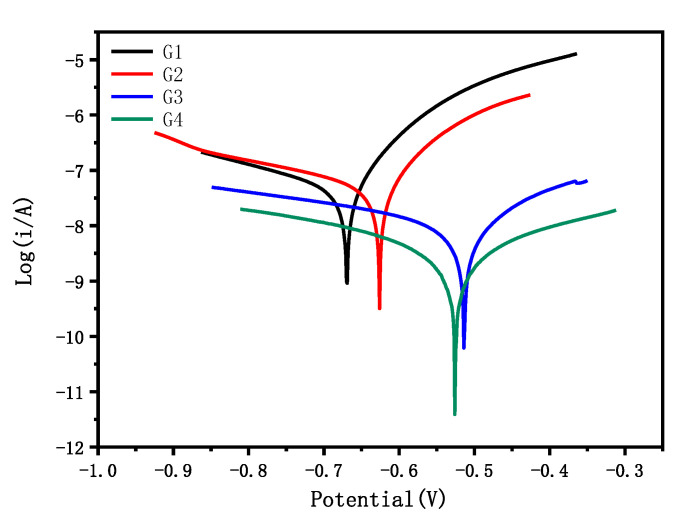
Each coating’s Tafel curve after immersion for 31 d.

Figure 8 shows the Tafel curve of carbon steel coated with G1, G2, G3 and G4 immersed in the simulated saline-alkali solution for 31 d. The coating’s anticorrosion performance is mainly evaluated by the corrosion current density I_corr_ and the corrosion potential E_corr_. Generally, the corrosion current density is compared first. If the corrosion current density is very close, the corrosion potential is further compared [27]. From Figure 8, we found that G4 had the smallest corrosion current density, and G1 had the largest corrosion current density.

Table 2 shows the corrosion potential E_corr_, corrosion current density I_corr_, polarization resistance R_p_ and coating protection efficiency η of each coating in the simulated saline solution after immersion for 31 d. Among them, G4 had the smallest corrosion current density, only 2.878 × 10^−9^ A/cm^2^, and the largest polarization resistance, reaching 1.475 × 10^7^ ohm, which was two orders of magnitude higher than G1. The corrosion current density of G3 and G2 was less than that of G1, the polarization resistance and protection efficiency were improved to different degrees, and the protection efficiency of G3 was 84.44%. The corrosion potential of G4 was −0.527 V, and the protection efficiency was as high as 95.76%, which had obvious advantages compared with other coatings [28].

### 3.4. Mott–Schottky Curve Analysis

**Figure 9:**
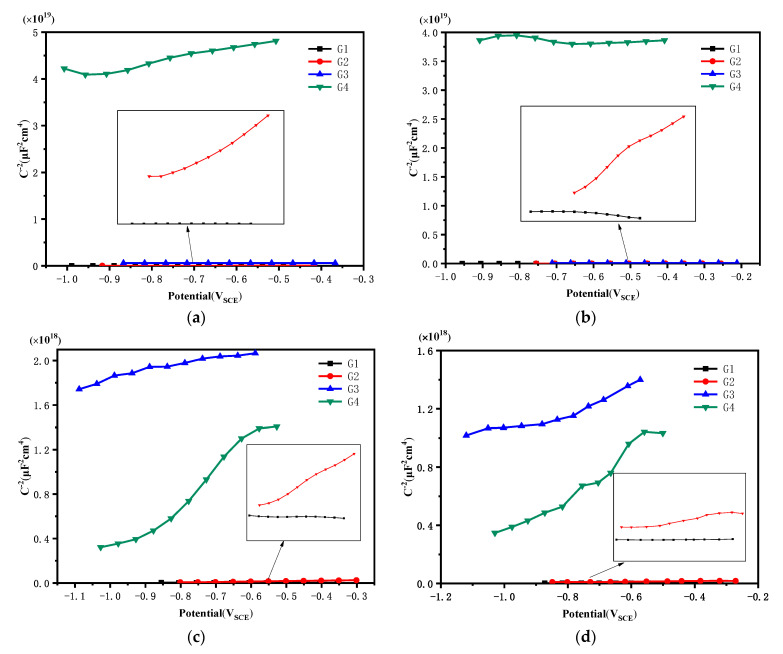
Each coating’s Mott–Schottky curve after immersion: (**a**) 1 d, (**b**) 11 d, (**c**) 21 d, (**d**) 31 d.

Figure 9 shows the Mott–Schottky curve of G1, G2, G3 and G4 immersed in the simulated saline-alkali solution. It can be seen from Figure 9 that when immersed for 1 d, the Mott–Schottky curves’ slopes of each coating were all positive, which conformed to the properties of n-type semiconductors. G4 had the largest slope, so its carrier density was the smallest, and the electron removal and ion breakthrough inside the coating were also the least. The slope of the Mott–Schottky curve of G3 was greater than that of G1 and G2, therefore, G3 had a relatively small carrier density. Since GO was easy to stack between the layers in the epoxy resin, it led to the agglomeration of GO in the epoxy resin. Poor dispersibility led to uneven coating. The weakened coating was the same as the EP coating, so its anticorrosion performance was poor. The EP coating had poor anticorrosion effect due to the lack of fillers, and its internal carrier density was also the largest. Table 3 shows each coating’s carrier density after immersion for 1 d, 11 d, 21 d and 31 d. With the lengthening of the immersion time, the carrier density of G1 and G2 gradually increased, while the corrosive medium continued to penetrate into the coating, and the corrosion continued to intensify. The carrier density of G4 increased slowly and remained at the order of magnitudes of 10^12^ for 31 d. This was because the penetration rate gradually decreased with the increase of the moisture content of the coating during the infiltration of water molecules into the coating, and the water solubility of the CeO_2_ nanoparticles hindered the erosion medium to a certain extent [29]. The carrier density of G3 was 5.530 × 10^15^ cm^−3^ after immersion for 31 d. It could be seen that the anticorrosion performance of G3 decreased significantly with the increase of the immersion time, and the later performance was close to G1 and G2.

## 4. Corrosion Morphology Comparison

### 4.1. Corrosion Depth Analysis of Substrate

**Figure 10:**
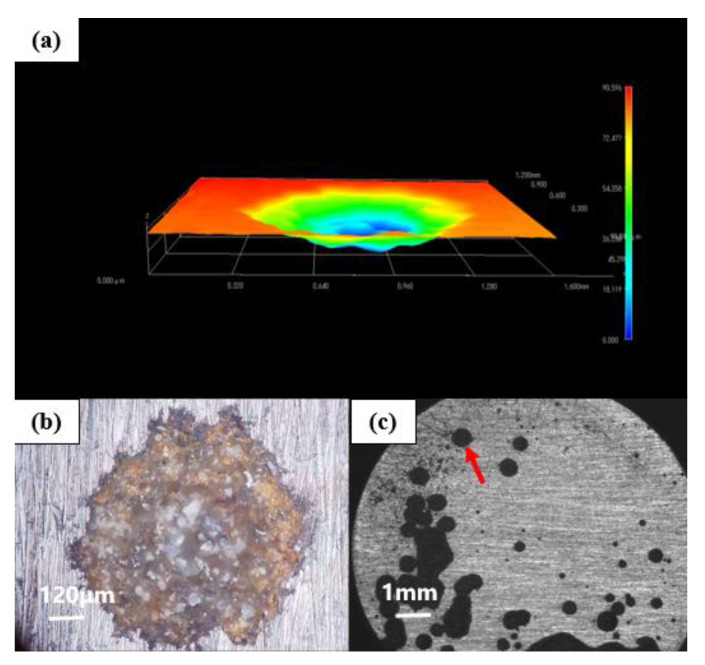
Metal substrate of G1 immersed for 31 d: (**a**) 3D stereo microscope image; (**b**) 3D-fitted morphology; (**c**) stereo microscope image.

Figure 10 shows the morphology of G1-coated substrate soaked in the simulated saline-alkali solution for 31 d under a stereological microscope. The corresponding 3D topography shows that the corrosion depth of the G1-coated substrate was 90.596 μm and showed serious corrosion. The corrosive medium continuously reached the metal substrate through the micropores in the coating. The corrosion gradually expanded from one point to the inside to form deeper pitting corrosion. Furthermore, corrosion pits spread to the surroundings, and a large area of corrosion appeared. There were many corrosion pits connected, indicating that the protection ability of G1 was weak and could not effectively prevent the attack of corrosive medium to the substrate.

**Figure 11:**
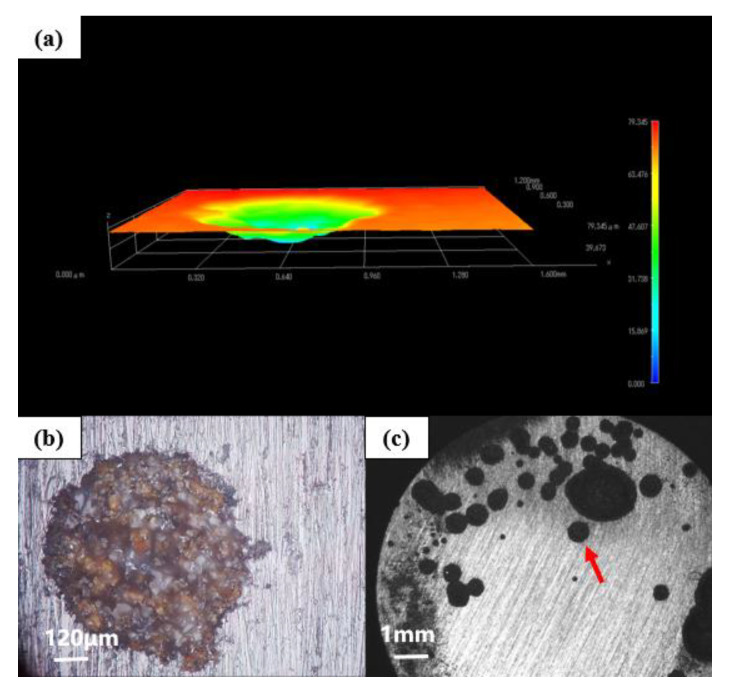
Metal substrate of G2 immersed for 31 d: (**a**) 3D stereo microscope image; (**b**) 3D-fitted morphology; (**c**) stereo microscope image.

Figure 11 shows the morphology of the G2-coated substrate soaked in the simulated saline-alkali solution for 31 d under a stereological microscope. The corresponding 3D morphology shows that the corrosion depth of the G3-coated substrate was 79.345 μm. There were many connected holes and a large area of pit corrosion in the metal matrix. It can be seen that the corrosion of the G2-coated substrate was concentrated, and most of it diverged from one point to the surrounding areas, indicating that GO cannot be well dispersed in the epoxy resin. The GO itself was stacked and the layers cannot be fully unfolded. In addition, its barrier efficiency to corrosive media was also reduced, resulting in concentrated and severe rust at the weak points of the coating.

**Figure 12:**
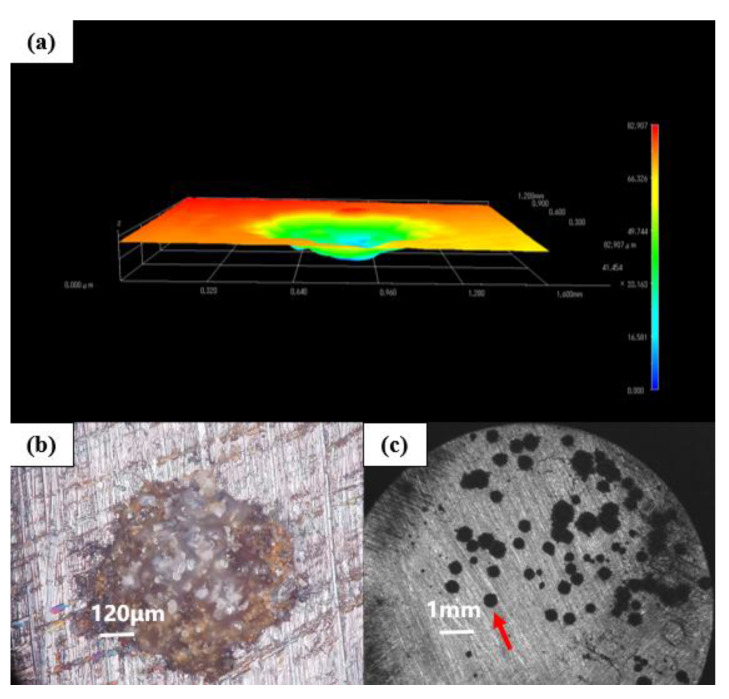
Metal substrate of G3 immersed for 31 d: (**a**) 3D stereo microscope image; (**b**) 3D-fitted morphology; (**c**) body view microscope image.

Figure 12 shows the morphology of the G3-coated substrate soaked in the simulated saline-alkali solution for 31 d under a stereological microscope. The corresponding 3D topography shows that the corrosion depth of the G3 coated substrate was 82.907 μm, which was smaller than that of G1 and G2. Compared with the above electrochemical test, it can be seen that the protection effect of G3 on the substrate decreased significantly with the extension of immersion time. On the one hand, there were many gaps between the CeO_2_ particles, so that there were still more transmission channels for the erosion medium. On the other hand, because the limited CeO_2_ particles had a hydrolysis limit, with the continuous infiltration of the erosion medium, the generated precipitation would no longer increase [21]. Therefore, its shielding effect on corroding ions lacked long-term performance. It had a certain barrier ability to corrosive media in the early stage, and its corrosion resistance was insufficient in the later stage, which led to more serious corrosion of the substrate.

**Figure 13:**
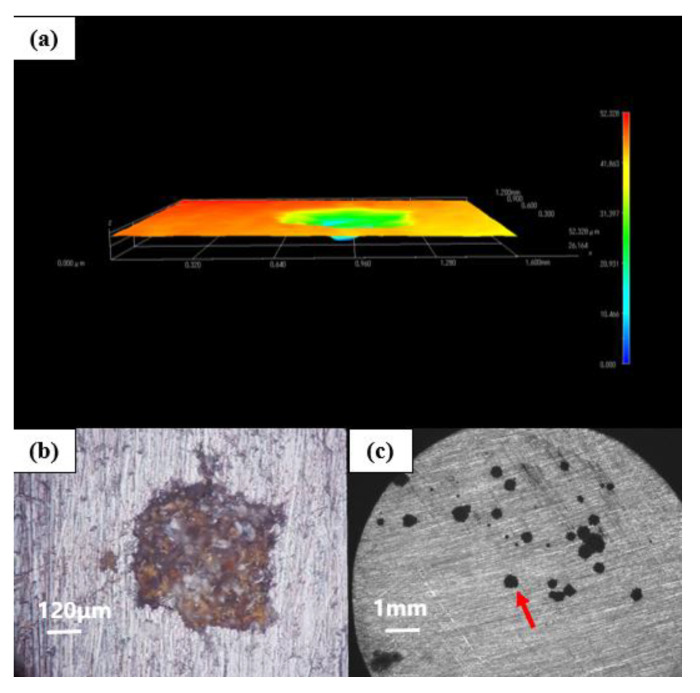
Metal substrate of G4 immersed for 31 d: (**a**) 3D stereo microscope image; (**b**) 3D-fitted morphology; (**c**) stereo microscope image.

Figure 13 shows the morphology of the G4-coated substrate soaked in the simulated saline-alkali solution for 31 d under a stereological microscope. The corresponding 3D topography shows that the corrosion depth of the G1-coated substrate was 52.328 μm, which was the smallest among all coatings. Moreover, there were pitting corrosion or corrosion pits occurring on the metal matrix, and the corrosions were discrete points mostly without large-scale connection. The diameter of the corrosion pits was the smallest, showing that the coating had good anticorrosion effect and effectively delayed the erosion of the substrate by the corrosive medium, which provided excellent protection for the metal substrate.

### 4.2. Corrosion Area Analysis of Substrate

**Figure 14:**
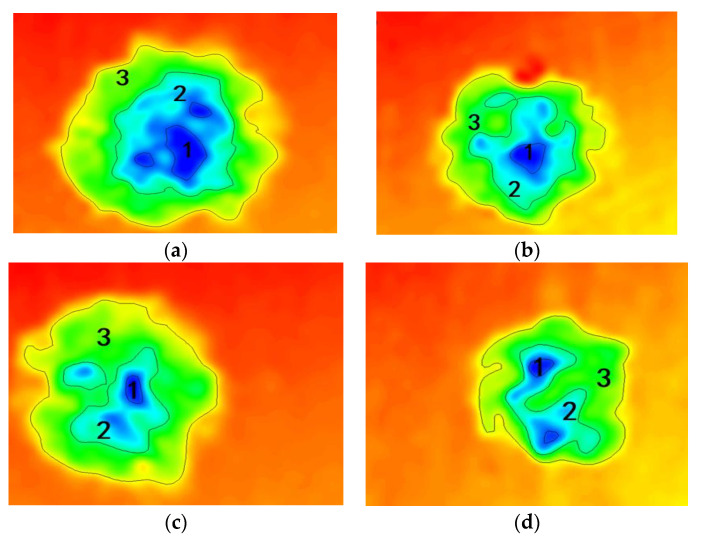
Body microscopic corrosion analysis of each coating’s substrate immersed for 31 d: (**a**) G1; (**b**) G2; (**c**) G3; (**d**) G4.

**Figure 15:**
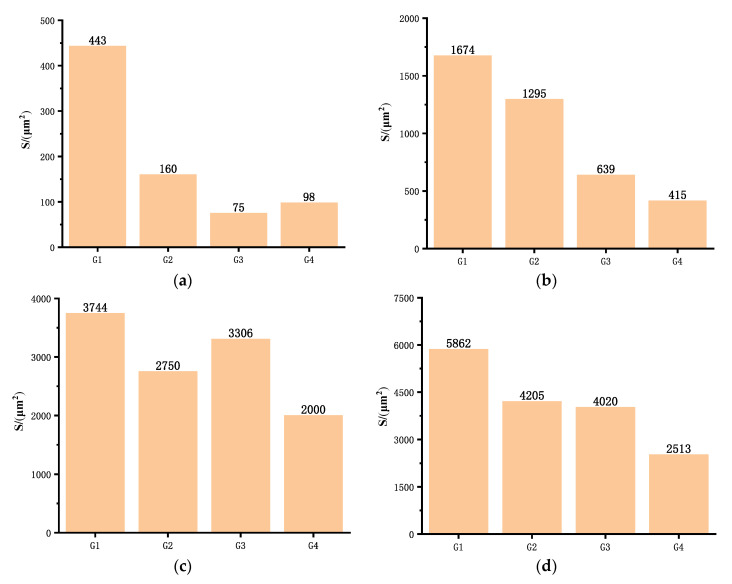
Corrosion area analysis of each coating’s substrate immersed for 31 d: (**a**) area 1 of corrosion, (**b**) area 2 of corrosion, (**c**) area 3 of corrosion, (**d**) total area of corrosion.

Figure 14 and Figure 15 show the body microscopic corrosion analysis and corrosion area analysis of each coating’s substrate immersed for 31 d. Table 4 shows the results of the analysis of the corresponding corrosion areas in Figure 12. The total corrosion area of G4 was 2513 μm^2^, which was the smallest among the four groups of coatings. The corrosion area 1 of G3 was 75 μm^2^, which was smaller than that of G4. This was because the latter corrosion pit was actually the result of the development of two pitting corrosions. There was no connection between the two pits in the bulk, but only on the surface of the substrate. Furthermore, the single corrosion pit area of G4 was smaller than that of G3, and the total corrosion area of the former was much smaller than the latter. This is because the lamella structure of GO was stretched by CeO_2_, which improved the interception efficiency of the corrosive medium. At the same time, the hydrolysis of CeO_2_ particles blocked the transmission channel of the corrosive medium in the coating. Therefore, even if some corrosion occurred on the metal substrate, the accumulation of subsequent corrosion products and the blockage of the transmission channel would greatly reduce the development speed of corrosion. The total corrosion area of G2 was close to that of G3. That was because the corrosion products of the former accumulate on the substrate, and the stacking of GO limited the corrosion development speed to a certain extent. However, the deepest corrosion area of the two were quite different. Because the former started to corrode earlier, its corrosion depth and corrosion area were larger, while the latter corroded relatively late. Therefore, it had better protection performance than G2. This also showed that smaller size and better dispersion were of great significance to the corrosion resistance of coatings when nanomaterials were used.

## 5. Conclusions

In this paper, the anticorrosion performance of a CeO_2_–GO (4:1)/EP coating during immersion in a simulated saline-alkali solution was studied. The EP coating, GO coating, CeO_2_ coating and CeO_2_–GO (4:1)/EP coating were prepared for the comparative study. The following conclusions can be obtained based on our experimental evidence.

(1) The anticorrosion properties of CeO_2_–GO nanocomposites as a coating were analyzed by OCP, EIS, Tafel curve and Mott–Schottky curve. The corrosion current density of the CeO_2_–GO (4:1)/EP coating after immersion for 31 d was the smallest of the four coatings, only 2.878 × 10^−9^ A/cm^2^, which was nearly two orders of magnitude lower than the EP coating. The corrosion current density of the CeO_2_/EP coating was 1.056 × 10^−8^ A/cm^2^, and the protection efficiency was 84.44%, which was also greatly improved compared with the EP coating, benefiting from the dense filling effect of CeO_2_ nanoparticles.
(2) The 3D fitting image analysis with the stereo microscope showed that the CeO_2_–GO (4:1)/EP coating had the best barrier effect on the corrosive medium, and the depth of pitting corrosion was the smallest. By analyzing the area of different corrosion depths, the total corrosion area of the CeO_2_–GO/EP coating is 2513 μm^2^, which was much smaller than that of the EP coating, GO/EP coating and CeO_2_/EP coating, indicating that CeO_2_–GO (4:1) nanocomposite limited the further development of corrosion.
(3) The product formed by the hydrolysis of CeO_2_ can fill the micropores on the surface and internal capillary channels of the coating and improve the compactness of the coating. At the same time, the CeO_2_–GO (4:1) nanocomposite had good compatibility with EP and could still show good anticorrosion performance in saline-alkali and temperature changing environments.

## Figures and Tables

**Table 1:** Solute mass in simulated saline solution.

Component	NaHCO_3_	CaCl_2_	Na_2_SO_4_	MgCl_2_	NaCl
Concentration (g/L)	0.113	0.232	2.5304	0.5698	3.1069

**Table 2:** Kinetic parameters of each coating in simulated saline-alkali solution after immersion for 31 d.

Coating	E_corr_ (V)	I_corr_ (A/cm^2^)	η (%)	R_p_ (ohm)
G1	−0.669	6.788 × 10^−8^	-	4.985 × 10^5^
G2	−0.631	5.783 × 10^−8^	14.81	5.078 × 10^5^
G3	−0.514	1.056 × 10^−8^	84.44	4.093 × 10^6^
G4	−0.527	2.878 × 10^−9^	95.76	1.475 × 10^7^

**Table 3:** Each coating’s carrier density after immersion for 1 d, 11 d, 21 d and 31 d.

Coating	1 d N_D_ (cm^−3^)	11 d N_D_ (cm^−3^)	21 d N_D_ (cm^−3^)	31 d N_D_ (cm^−3^)
G1	9.484 × 10^14^	7.349 × 10^15^	9.039 × 10^16^	4.243 × 10^16^
G2	8.923 × 10^14^	2.327 × 10^15^	9.039 × 10^15^	9.289 × 10^15^
G3	1.494 × 10^13^	1.664 × 10^13^	1.808 × 10^14^	5.530 × 10^15^
G4	5.970 × 10^11^	3.56 × 10^12^	5.165 × 10^12^	9.088 × 10^12^

**Table 4:** Results of the analysis of the corresponding corrosion areas in Figure 12.

Coating	Corrosion Area (μm^2^)			
	1	2	3	Total
G1	443	1674	3744	5862
G2	160	1295	2750	4205
G3	75	639	3306	4020
G4	98	415	2000	2513

## Data Availability

Not applicable.
